# Delivery of mRNA Therapeutics Beyond Infectious Diseases: Design Innovations and Applications in Oncology, Cardiovascular, and Rare Genetic Diseases

**DOI:** 10.3390/ph19050663

**Published:** 2026-04-24

**Authors:** Snehitha Akkineni, Mahek Gulani, Samir A. Kouzi, Martin J. D’Souza, Mohammad N. Uddin

**Affiliations:** 1 College of Pharmacy, Mercer University, Atlanta, GA 30341, USA; snehitha.akkineni@live.mercer.edu (S.A.); mahekanil.gulani@live.mercer.edu (M.G.); dsouza_mj@mercer.edu (M.J.D.); 2 School of Pharmacy, Wingate University, Wingate, NC 28174, USA; skouzi@wingate.edu

**Keywords:** mRNA, mRNA therapeutics, lipid nanoparticles, design principles, nanocarrier engineering

## Abstract

Empowered by nanotechnology, messenger RNA (mRNA) therapeutics have shown a rapid evolution post COVID-19 from a conceptual platform to a clinically validated modality, and they diversified into oncology, cardiovascular diseases, and rare disorders. As a template for in situ protein production, it offers several advantages over traditional proteins and DNA drugs. The intrinsic stability of mRNA and its sensitivity to innate immune sensing hinder its capacity for immediate cellular entry, necessitating its need for a delivery system to obtain optimal therapeutic potential. This review explores the innovations in nanocarrier engineering, design principles for lipid nanoparticles-mRNA (LNPs) platforms, and their clinical translation across the prominent indications. It also addresses their safety, immunogenicity, and scalability while addressing the key limitations and manufacturing scalability through comparative platform analysis. Although LNPs usually dominate their delivery through encapsulation and manufacturability, their limitations, like repeat dose reactogenicity and liver tropism, require next-generation designs like SORT lipids, stimuli-responsive hybrids for extrahepatic targeting. In oncology, LNP-mRNA drives the neoantigen vaccines, and rare diseases leverage the transient enzyme replacement. While the safety profiles highlight the innate immune tuning through nucleoside mods and lipid biodegradability, chronic administration risks are still persistent. While there are novel scalability options like microfluidic mixing to support the production gaps in organ selectivity and durability, their adoption is hindered. We outline the future directions to perceive mRNA’s full potential as a broader therapeutic class.

## 1. Introduction

Messenger RNA (mRNA) therapeutics are considered a paradigm shift in modern therapeutics, as they can address current unmet needs in vaccines, oncology, cardiovascular diseases, and rare genetic disorders. Driven by nanotechnology, specifically lipid nanoparticles (LNPs), this line of therapeutics circumvents the longstanding barriers and offers rapid adaptability without posing any genomic integration risks [1,2,3]. Amongst the several delivery techniques, polymeric nanoparticles, cationic liposomes, and hydrogel depots, LNPs are the most prominent platform for systemic and intramuscular administration of mRNA. They outperform the rest based on their potency and safety profile. Several reviews examine the compositional aspects of LNPs; however, the nanoscale structural developments and their material properties are not covered. Dating to 1961, ever since the discovery of mRNA, its therapeutic potential has remained dormant for several decades because of its instability, limited cellular uptake, and its potent innate immunogenicity through Toll-like receptor activation. Barriers to clinical translation persisted into the early 2000s, when ionizable lipids were developed for pH-responsive LNPs to encapsulate mRNA and minimize toxicity [4]. The development of mRNA therapeutics has also been inclined with the rethinking of nanocarrier engineering. Early on, the nanocarrier systems were mostly relying on the optimization of lipid ratios, particle sizes, and encapsulation efficiencies. However, with the latest advances, these have shifted towards incorporating more biomimetic, stimuli-responsive elements for enhancing the intracellular trafficking and tissue-specific biodistribution. These developments state that the therapeutic potential of mRNA is interlinked with the behavior of its carrier [5,6]. These engineering techniques are also strengthened by the advances in microfluidics, nanoprecipitation, and automated formulations to reduce the dependency on empirical optimization and further enhance the reproducibility [7].

Recently, mRNA therapeutics have also been expanded into oncology, cardiovascular disease, and rare genetic disorders, as there is a growing need for delivery systems that are able to navigate the complex tissue microenvironments. Unlike the regular vaccines, which target antigen-presenting cells, mRNA therapeutics require precise delivery to tissues with altered physiological states [8]. This diversification has prompted the reevaluation of therapeutic delivery challenges that are beyond pharmacokinetics. Oncology, for example, needs efficient delivery, modulation in the tumor microenvironment, overcoming immune suppression, and coordinating with the current treatment modalities, such as checkpoint inhibitors [9]. For cardiovascular applications, nanocarriers must withstand the shear forces and rapid clearance, as there are endothelial dysfunction and altered hemodynamics, which complicate the delivery. And in terms of rare genetic disorders, they require delivery of therapeutic agents in tissues that have limited regenerative capacity [10,11]. With the extensive requirements for each category, the therapeutic contexts highlight the need for systems that can be adaptable and support repeated dosing without eliciting adverse immune responses. Innovations like selective organ targeting lipids and hybrid nanocarriers can address the research gaps, as they have the potential for navigating the immunological barriers effectively. The extrahepatic delivery still remains inefficient for several organs, similar to the invasive route of administration or high systemic doses, as there is a constraint with both patient compliance and therapeutic index [12].

Parallel to these advancements, the field is also undergoing a shift in the manufacturing of mRNA therapeutics post pandemic. With COVID-19, there were critical vulnerabilities in global mRNA production, such as dependence on specialized lipids, insufficient surge capacity, and modularity in production, highlighting the need for manufacturing frameworks that meet the need with no extensive formulation. Several studies have emphasized that modular nano assembly allows the integration of mRNA as a common workflow, enhancing the scalability of the field [13]. It is also necessary that there is a development in computational tools along with the manufacturing and regulatory conditions to predict the nanoparticle behavior, to optimize the mRNA structure, and to model the intracellular trafficking pathways. For this, machine learning and high-throughput screening platforms can be used to map the relationships between the nanocarrier composition, biological outcomes, and rational design strategies. With the automated formulation, computational modeling, and data-driven optimization, the LNP mRNA field will be more integrated and predictive [14,15].

Combined, these developments illustrate the transition of the field from single-use vaccines to a more diversified therapeutic platforms that are not simply laboratory innovations but are more scalable and globally deployable technologies. Against this backdrop, the present review focuses on the nanotechnology-enabled mRNA therapeutics, emphasizing nanocarrier designs across lipids, hybrid, and smart systems. We have focused particularly on the design concepts that link the physicochemical properties of nanocarriers for next-generation mRNA therapeutics. Additionally, the manufacturing scalability that recognizes the industrial feasibility is also discussed, as it plays a major role in transforming the concept from bench to bedside. Through this, we aim to illustrate the current technologies’ position towards their translation and their scientific gaps, thereby providing insights into the challenges that need to be addressed.

## 2. Design Principles for Nanotechnology-Enabled mRNA Delivery

Development of such delivery systems involves several interconnecting principles, such as carrier composition and the functionality behind cellular uptake, along with their biodistribution. For optimal mRNA delivery, maximized transfection efficiency alone is inadequate; tailored organ tropism, immune activation, and biodegradation are also crucial when working with lipid nanoparticles (LNPs), lipid-polymer hybrids, and dendrimer-based carriers (Table 1) [16,17,18].

### 2.1. Ionizable Lipid Chemistry and Structure-Activity Relationships

Ionizable lipids play a major role as the central design element that aids in the cytosolic transport. An ideal ionizable lipid with linker chemistry, hydrophobic tails, and headgroup structure would determine the potency and toxicity. With pKa values ranging from 6.0 to 6.8, the lipids would remain neutral in the physiological pH, which allows them to reduce hemolysis and complement activation. These would also aid in the endosomal disruption, leading to the mRNA release. Based on the structure-activity relationships, branching, saturation, and ester linkages within the hydrocarbon tails modulate the biodegradability, thereby generating a huge impact on both efficacy and clearance [19,20]. According to Sabnis et al., the introduction of the biodegradable ester linkers to amino lipids enables mRNA delivery with enhanced tolerability and also supports repeat dosing in non-human primates, in turn highlighting the importance of biodegradability as an important design principle for chronic administration [21]. Moreover, Maier et al. have demonstrated biodegradable lipids that maintained high expression and simultaneously reduced long-term accumulation, thus supporting the principle of lipid scaffolding engineering with efficient hepatic and systemic clearance [22]. Based on these studies, it is evident that ionizable lipids that are designed as transient scaffolds demonstrate strong endosomal escape and mRNA release while undergoing the necessary metabolic breakdown to minimize the systemic burden. Despite the extensive SAR studies the endosomal escape and cytosolic stability remain unclear for the ionizable lipid mediated mRNA delivery.

### 2.2. Role of Helper Lipids

In addition to the ionizable lipid, structural phospholipids, sterols, i.e., cholesterol, and polyethylene glycol (PEG) lipids also play a major role in determining the morphology, stability, and in vivo behavior of the nanoparticles. PEG lipids can be defined as the conjugated lipids that are used on the surface of the LNPs for enhancing the colloidal stability, reducing the aggregation, and improving the circulation time. It is the steric stabilizer in the nanoparticle shell [23]. Certain phospholipids also influence how the LNPs promote non-bilayer structures, which facilitate the membrane fusion and endosomal escape. Cholesterol, however, modulates the membrane rigidity and packing [3,24]. Moreover, PEG lipids are critical for controlling the colloidal stability and protein adsorption while also balancing the cellular uptake with their design. Mui B.L. et al. have shown that the PEG lipids with faster desorption show elevated siRNA activity in vivo, where the PEG is allowed to shed post injection, which then exposes the particle surface for any cell interactions while simultaneously preventing aggregation in the formulation. Thus, it is necessary to understand the PEG-lipid desorption kinetics and tune them according to the route and target tissue so there will be faster shedding to achieve rapid local uptake and slower shedding in systemic formulations that are long-circulating [25]. Sahay et al. have emphasized that the nanomedicines face sequential barriers such as cellular uptake, endosomal trafficking, and endosomal escape, where each of them require their specific design solutions [26]. While Gilleron et al. have used image-based analysis showing that only a small fraction of siRNA-LNPs that are internalized usually escape the endosomal pathway for reaching the cytosol, thus making endosomal escape efficiency a key rate-limiting step for the functional delivery [27].

### 2.3. Formulation Composition and High-Throughput Optimization

In terms of formulation composition, the ratios of ionizable lipids, phospholipids, PEG lipid, and cholesterol critically define the particle size, encapsulation, and biodistribution. According to Cheng et al., minor changes in the lipid composition enable the tissue-specific mRNA delivery with LNPs; the slightest tweaking of molar ratios has altered the organ targeting and cellular delivery without changing any particle size or charge. This states the underlying importance of systematic design of experiments for mapping the formulation space rather than the trial-and-error method. With high-throughput in vivo screening of over 100 LNPs, several identified formulations selectively delivered mRNA to endothelial cells [28]. Following the design of experiments, Kauffman et al. have employed fractional factorial and definitive screenings to identify the optimal combinations that have maximized the mRNA expressions in vivo, thereby demonstrating that the microfluidic formulations with LNPs can be optimized with statistical DOE approaches. Specifically, the variation in the lipid ratios and structures showed a 7-fold potency increase for the erythropoietin-mRNA in the liver with the addition of higher ionizable lipid-to-mRNA ratios [29].

### 2.4. Delivery Principles Based on Route-Specific and Organ-Selective

mRNA delivery can be route-specific and organ-dependent. Based on Hassett et al., the LNPs optimized for intravenous siRNA delivery were unsatisfactory for intramuscular (IM) mRNA vaccines, due to which an IM-specific screening for biodegradable ionizable lipids was needed to generate formulations with enhanced tolerability and immunogenicity in rodents and non-human primates. However, the LNP-induced innate immune activation did not correlate with the strong vaccine responses, promoting the need for controlled stimulation. They have also shown that the vaccine LNPs can be reformulated for enhanced tolerability without compromising the immunogenicity and decoupling the innate reactogenicity from the adaptive responses [30]. This was further advanced by Cheng et al., using Selective Organ Targeting (SORT) nanoparticles that were altered by the addition of charged lipids at 1–2 mol%, which reprogrammed the liver-tropic LNPs to either lung or spleen without any change in the particle size, thereby strongly establishing a modular strategy for tropism tuning [31]. For non-parenteral routes an inhalable dry powder product (DPP) of mRNA-LNPs for pulmonary delivery was developed by Sarode et al. They have shown that the excipients like leucine and mannitol, along with the spray drying technique, preserve and protect the nanoparticle integrity along with the mRNA activity through aerosolization and rehydration, highlighting the manufacturability and solid-state stability as critical designs for inhaled formulations [32].

### 2.5. Immunological Constraints, Including Safety and Tolerability

It is important to note that the safety and tolerability constraints play a major role in design choice. As noted by Sabnis et al. and Maier et al., incorporating biodegradable motifs into ionizable lipids supports reducing chronic exposure, which is essential for therapeutic non-vaccine mRNA applications [21,22]. The immunogenicity of both lipid components and mRNA should be co-optimized, with proper attention to complement activation, cytokine profiling, and off-target organ injury. Based on these studies, the effective nanotechnology-enabled mRNA delivery systems are not usually defined by a single “best” lipid or its composition, but rather by their specific designs, ionizable lipid chemistry, helper components, PEG kinetics, route, and organ targeting, along with their optimized safety.

**Table 1:** Core design principles for mRNA nanocarrier engineering.

Design Principle	Advantages	Limitations	Research Needs	References
Ionizable Lipids	Linker chemistry, degree of branching, hydrophobic tails	Branching, ester hydrolysis impacts storage stability	Chronic dosing data, comprehensive SAR databases for comparing tail chemistry to clearance	[21,22,25]
Helper Lipids	Promote non-bilayer structures; cholesterol modulates rigidity	Stability, Uptake Inhibition and desorption kinetics are route dependent	PEG Shedding assay and lipid libraries with tissue specificity information	[33,34]
Formulation Optimization	Organ targeting with optimized molar ratios Design of Experiments (DOE) screening fastens discovery	High throughput screening limited to liver/lung models Translation is uncertain	Disease-specific formulation and DOE for rare diseases	[33,35]
Route- And Organ Specific Design	Retarget LNPs, decouples potency from innate immunity	Non parental routes underexplored	Cross comparative studies with other routes and aerosol performance metrics	[18]

### 2.6. Analytical Characterization

Certain analytics quantitatively link the formulation design to its performance, where the design metrics involve lipid ratios, pKa, and nitrogen to phosphate ratio. Moving on to the physicochemical profiling, size, encapsulation efficiency, and morphology determination are important, as is the functional validation (Table 2). The thioflavin T titration quantifies the protonation efficiency, which directly predicts the endosomal escape potential. The cryo-transmission electron microscopy visualizes the formation of hexagonal lipid phases under acidic conditions [36].

## 3. Innovations in Nanocarrier Engineering

### 3.1. Overview of Current and Emerging Nanocarrier Platforms

**Figure 1:**
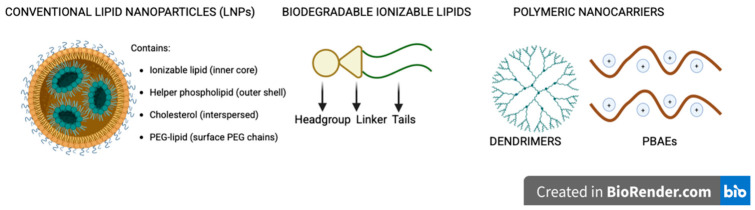
Depiction of the conventional and emerging nanocarrier platforms. Created in BioRender. Akkineni, S. (2026) https://BioRender.com/4ohhmzd, accessed on 20 April 2026.

The current nanotechnology platforms that are used for mRNA delivery are dominated by lipid nanoparticles (LNPs) (Figure 1). They are the non-viral mRNA carriers that contain an ionizable lipid, helper phospholipid, and cholesterol. Together, they protect the mRNA from degradation, thereby promoting cellular uptake and facilitating endosomal escape. Conventional LNPs still have certain important limitations, such as liver accumulation, tissue specificity, and dose-dependent immunogenicity with repeated dosing. Moreover, the cationic lipid and polymer systems can sometimes show toxicity and serum instability through in vivo performance. The immunogenicity and biodistribution constraints have motivated the development of next-generation carriers as shown in Figure 1 [40,41,42]. Nanocarrier engineering has transformed mRNA delivery because it addresses several key limitations of conventional lipid nanoparticles, including weak tissue targeting, immunogenicity, and challenges in large-scale manufacturing. In terms of lipid innovations, biodegradable ionizable lipids and charge-switching systems represent pivotal advancements in lipid nanoparticle (LNP) formulations for mRNA therapeutics, addressing both endosomal escape and targeted delivery. These systems optimize the LNP performance through pH-responsive ionization for cytosolic release, along with the incorporation of cleavable bonds that mitigate accumulation and immunogenicity during repeated dosing [43]. Non-lipid polymeric systems include dendrimers, poly(β-amino esters), and polysaccharide carriers. They offer several alternatives to LNPs for mRNA therapeutics, thereby providing tunability, low immunogenicity, and custom targeting. Hybrid and biomimetic platforms include exosome-like vesicles and cell membrane-coated nanoparticles. Smart stimuli-responsive carriers integrate endogenous triggers. Collectively, these novel engineering strategies expand mRNA applications beyond its applications in oncology, autoimmune disorders, and regenerative medicine, thus accelerating its structure-activity optimization to enhance its clinical progression [44].

### 3.2. Innovations in Lipid-Based Carriers

There is a surge in the development of biodegradable ionizable lipids, which provide leverage for modular synthesis and accelerate structure-activity relationship (SAR) exploration. A study by Xu et al. has introduced the Passerini three-component reaction (P-3CR) platform, which enables a catalyst-free one-pot assembly of 144 diverse lipids with amine headgroups, isocyanides, and aldehydes that yield acylamide scaffolds with hydrolyzable ester bonds for rapid enzymatic degradation. With high-throughput screening, A4B4-S3 was identified, and it features a dimethylamino propionic acid headgroup, both branched and unsaturated tails, along with optimal methylene spacers. This has outperformed the SM-102 in intramuscular luciferase expression [45]. Han et al. have used a two-component Michael addition between amines/thiols and dialkyl maleates for assembling the ionizable lipids in one catalyst-free, minimal-solvent step. This catalyst-free strategy yields ionizable lipids with high atom economy along with great product yield, thus identifying lead candidates that outperform the benchmarked lipids in delivering the CRISPR-based mRNA therapeutics in vivo [46]. Chen et al. introduced a disulfide-bond-bridged ester linker for synthesizing a library of 96 linker-degradable ionizable lipids having cone-shaped architectures that enhance endosomal escape and rapid mRNA release. The high-performance ionizable lipids, which are linker-degradable, have improved mRNA delivery in vivo, thus enabling tumor targeting along with improved cancer metastasis delineation after they were administered intraperitoneally [47].

### 3.3. Charge-Switching and Stimuli-Responsive Lipids

Charge-switching systems are novel in nanocarrier engineering, which refine the delivery through enabling environment-responsive transitions, including enzyme or stimulus-triggered shifts that change from negative to positive zeta potentials. The enzyme responsive LNPs were designed as cationic lipid nanoparticles coated with polyphosphates, delivering a negatively charged zeta potential at baseline, and once they undergo dephosphorylation, there will be a charge conversion by the intestinal alkaline phosphatase (IAP) [48]. Following this, the microfluidics technique was used to prepare the LNPs, which underwent charge conversion from negative to positive, thereby enhancing the mucus diffusion, cellular uptake, and transfection in intestinal tumor cell lines like Caco-2 [49]. The charge-altering LNPs that are hydrogen peroxide responsive were engineered through incorporating phenylboronic acid (PBA) residues in the tertiary ammonium headgroup of the ionizable lipid so that the PBA cleavage by H_2_O_2_ reduces surface charge density and promotes the siRNA release. Moreover, under physiological pH conditions in the presence of H_2_O_2_, removal of the PBA groups facilitated lysosomal escape and enabled tumor-selective gene silencing following in vivo siRNA delivery [50].

### 3.4. Polymeric Nanocarriers

Because of the branched architecture that enables high charge density for mRNA condensation and considerable endosomal escape, dendrimers outperform the conventional carriers. Based on a study by Trines et al., they have enhanced the mRNA complexation and delivery to hematopoietic stem/progenitor cells in bone marrow through IV administration by screening eleven distinct dendrimer-based apolipoprotein nanoparticles and integrating the polyvalent ionizable cationic dendrimers into apolipoprotein nanoparticles. These have been shown to outperform the clinical ionizable lipids in protein expression and use safer natural materials [51]. Moreover, one-component ionizable amphiphilic Janus dendrimers (I-AJDs) were developed as synthetic and tunable alternatives to LNPs. Without any helper lipids, these enable the systemic delivery of TGF-β mRNA to the murine lung parenchyma. I-AJDs normally assemble themselves into mRNA nanoparticles, thus providing low immunogenicity and high mRNA encapsulation for organ-specific targeting [52].

Furthermore, the poly(β-amino esters) (PBAEs) enhance the hydrolysis and proton sponge-mediated escape by featuring the pH-responsive tertiary amines with a pKa ranging between 6.5 and 7.2. Kim et al. have developed a library of PBAE polymers through the integration of various amine and acrylate monomers, thus producing 55 distinct PBAE types. Further screening led to the identification of polymeric nanoparticles that have shown elevated mRNA expression efficiency and have also maintained the expression levels at the injection site for up to two weeks. These nanoparticles have not shown any liver toxicity, making them ideal for localized therapy [41]. Additionally, Yong et al. have designed “four-in-one” branched PBAEs (O-LhPAEs) by synthesizing poly(β-amino esters) with the combination of tertiary and quaternary amines, cholesterol-like moieties, zwitterionic groups, and hydrophobic alkyl tails. They have prepared 60 distinct O-LhPAE variants using the sequential Michael addition followed by the formulation of mRNA polyplexes. From this, 20%b-3C-2P12 has achieved 93.1% mRNA transfection across 11 different cell types, proving that the study has established a novel strategy to design the LNP-like polymer that is safe and efficient for mRNA delivery, particularly for systemic and nebulized applications [53]. Similarly, copolymers of lysine and isoleucine represent a promising class of amphiphilic cationic polypeptides that are necessary for mRNA delivery. A study by Pilipenko et al. has synthesized these copolymers via ring-opening polymerization of lysine and isoleucine and has optimized the ratio for achieving a small size and high encapsulation. These polyplexes have demonstrated enhanced protection against degradation compared to homopolymer poly(L-lysine). They also showed low cytotoxicity [54]. This positions the lysine and isoleucine as copolymers that are distinct from the conventional lipid-based systems, especially for applications that require tunable biodegradability. Polysaccharide carriers like chitosan and hyaluronic acid leverage the biocompatibility and mucoadhesion for mucosal/oral delivery. Garcia et al. have studied that through the optimization of ratios between chitosan-N-arginine (CSA) and DOTAP, high transfection efficiency and reduced cytotoxicity across different cell lines can be achieved, as this is crucial for the development of mRNA carriers. They noted that in the lipid formulations containing CSA, the physicochemical stability was enhanced while also improving the mRNA complexation; the transfection efficiency in HEK293T cells was also significantly high. In terms of cytotoxicity, CSA has reduced the toxicity, while DOTAP-based complexes have shown high toxicity [55].

### 3.5. Hybrid and Biomimetic Nanocarriers

Hybrid and biomimetic systems such as exosome-like vesicles (ELVs) and cell membrane-coated nanoparticles (CM-NPs) mark a fundamental shift in mRNA delivery by harnessing natural extracellular vesicle biology for overcoming the limitations of synthetic lipid nanoparticles, like poor targeting and inefficient endosomal escape. Exosome-like vesicles are engineered synthetic or cell-derived nanovesicles that recapitulate key properties of native exosomes, such as low immunogenicity, membrane-fusion-driven delivery, and intrinsic stability. Ginger-derived exosome-like nanoparticles (GELNs) are selectively internalized by the epithelial cells in the intestines and macrophages through caveolin-dependent endocytosis and micropinocytosis, which results in increased accumulation within the gut when compared with several synthetic nanoparticles. When GELNs are orally administered, their associated bioactivated lipids, along with miRNAs, tend to provide localized anti-inflammatory effects, including reduced pro-inflammatory cytokine production and enhanced mucosal integrity [56]. Tang et al. have developed inhaled mRNA nanoparticles with hyaluronic acid, which, when compared with regular cationic transfection reagents, claim that these mRNA-loaded formulations reach alveolar macrophages via the CD44 mediated uptake, showing higher transfection, thereby enhancing the intracellular delivery [57].

### 3.6. Comparison to Conventional Nanocarriers

All the above-described nanocarriers collectively address the performance gaps that limit the traditional LNPs and the cationic polymer systems (Table 3). The biodegradable ionizable lipids reduce the long-term retentions and enhance tolerability by overcoming the accumulation and repeated dosing challenges through the cleavable linkers and tail chemistries [16]. Charge-switching and stimuli-responsive designs have stronger control over the surface properties, penetration, and uptake when compared with the LNP formulations. Dendrimers and PBAEs offer delivery profiles that vary from the biased distribution of the LNPs. These functions are further extended by the hybrid and biomimetic carriers to improve the stability and tissue-specific uptake using natural membrane components and vesicle-like structures as leverage. All together, these innovations demonstrate the material designs that overcome the limitations of conventional nanocarriers for expanding the therapeutic reach [58,59]. Hybrid, and biomimetic platforms such as the exosome-like vesicles and cell membrane-coated nanoparticles usually extend the functionality beyond synthetic LNPs to improve the immune evasion and tissue-specific uptake [60]. Together, these platforms should not be seen as replacements for conventional LNPs but perceived as complementary designs that systematically address the limitations of traditional LNPs, hence expanding their therapeutic reach.

## 4. Therapeutic Diversification of LNP–mRNA Platforms

### 4.1. Oncology

**Figure 2:**
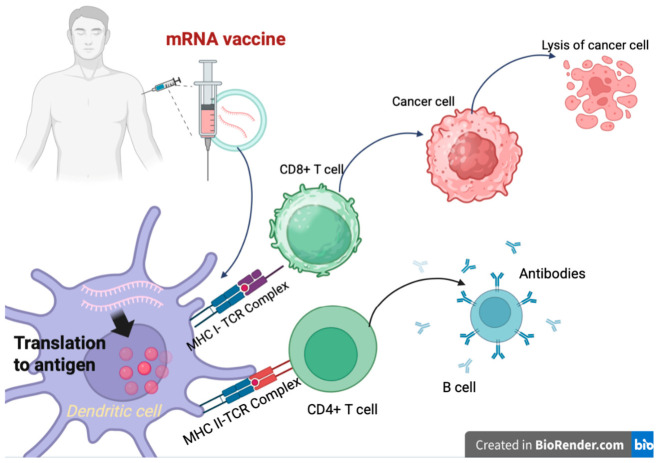
The immunological mechanisms that underlie mRNA-based tumor vaccines involve both humoral and cellular immunity responses in a sequential manner. After being administered, DCs internalize these vaccines, which are made of mRNA enclosed in lipid nanoparticles. The mRNA is translated into antigenic proteins inside the DCs, which are then delivered to T cells via the MHC I or MHC II pathways. Furthermore, the coordinated actions of cytokines further boost the cellular immunological mechanism. Crucially, the antigens that antigen-presenting cells (APCs) release could activate B cells, which then make neutralizing antibodies under the direction of CD4 T cells, strengthening the antitumor immune response. MHC-I presentation via proteasomal degradation and TAP-mediated transport, which activate CD8^+^ CTLs for direct tumor cell destruction, are the different sequential stages of the immune activation process. Created in BioRender. Akkineni, S. (2026) https://BioRender.com/ayqwel2, accessed on 20 April 2026.

The function of mRNA in oncology is to boost or increase the immune response against tumor antigens, in contrast to conventional vaccines that prevent infectious diseases. Different tumor antigens can be changed with the help of therapeutic mRNA flexibility [11]. Figure 2 depicts the immunological mechanisms via the MHC I and MHC II pathways which activate CD8^+^ and CD4^+^ T cells for a strong antitumor response in mRNA-based tumor vaccines. Tumor-associated antigens, which are found on both tumor cells and certain healthy cells, as well as tumor-specific antigens exclusive to cancer cells, like antiviral antigens and neoantigens resulting from tumor mutations, are important targets. Clinical trials are now testing several methods to prevent mRNA from being degraded, such as protamine-complexed mRNA, lipoplexes, lipid nanoparticles, and various dosing routes in diseases such as melanoma, prostate cancer, non-small cell lung cancer, and pancreatic ductal adenocarcinoma, which is one of the most difficult kinds [11]. While many trials are still in the early stages, several have progressed to phase 3 and demonstrated encouraging safety and efficacy outcomes. However, a fully universal mRNA-based cancer vaccine appears to be unattainable due to the complexity and variety of tumors, even within patients exhibiting the same subgroup of neoplasm. Personalized mRNA cancer vaccines that target tumor-specific neoantigens have gained popularity as a workable and promising remedy. Combining this kind of therapy with other treatments, including immune checkpoint inhibitors, could lead to novel oncology options.

mRNA-based cancer vaccines (Table 4) provide several benefits, including their quick and inexpensive manufacture, non-infectious nature, and good tolerability [69]. The intratumoral immune microenvironment must be well characterized to integrate it with current immunotherapies. Therefore, to induce antitumor responses, Fournier et al. tested nanostructured lipid carriers (NLCs), also known as Lipidots^®^, for delivering unmodified mRNA expressing Ovalbumen (OVA) antigen [70]. Results showed that the vaccine activated dendritic cells via Toll-like receptors (TLR) 4/8 and ROS signaling, induced antigen-specific T-cell responses, and showed strong preventive and therapeutic antitumor efficacy in vivo. Immunized mice exhibited intratumoral remodeling of innate and adaptive immunity with upregulated CD8^+^ T-cell-recruiting chemokines. Hence, Lipidots^®^ represented an effective mRNA cancer vaccine platform, and their combination with anti-PD-1 therapy enhances complete responses, overcomes tumor resistance, and promotes durable antitumor immune memory [70].

Li et al. reported a novel cancer immunotherapy in which intratumoral delivery of pathogen antigens via mRNA–lipid nanoparticles suppress tumor growth and prolongs survival across multiple solid tumor models. In mice pre-immunized with BNT162b2, intratumoral administration of the same vaccine tagged tumor cells with SARS-CoV-2 spike protein, rapidly recruiting pre-existing antiviral memory immunity to eliminate cancer cells and remodel the tumor microenvironment. This initial tumor clearance promoted antigen spreading and robust tumor-specific T-cell responses, leading to systemic antitumor immunity. Combination with anti-PD-L1 therapy further enhanced efficacy, including in immunologically “cold” tumors [71]. Ben-Akiva et al. developed biodegradable polymeric nanocarriers that enable ligand-free, systemic mRNA delivery to splenic dendritic cells. The platform overcomes challenges in DC transfection and lymphoid organ targeting and supports codelivery of mRNA and adjuvants to induce DC costimulatory signaling and antigen-specific CD8^+^ T-cell activation. This approach elicited robust antitumor immunity across multiple murine tumor models, validating systemically delivered polymeric mRNA cancer vaccines as a broadly applicable strategy [72].

### 4.2. Cardiometabolic Diseases

**Figure 3:**
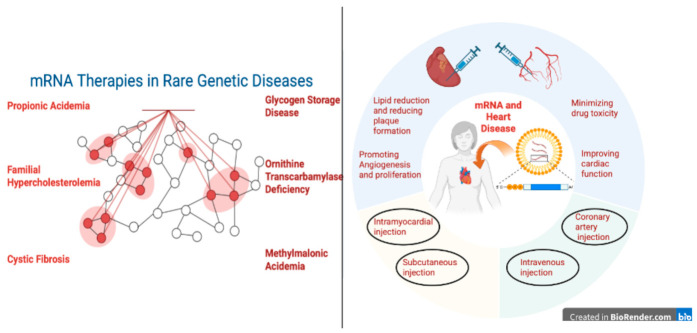
Schematic overview illustrating the expanding clinical scope of mRNA therapeutics beyond infectious diseases. Created in BioRender. Akkineni, S. (2026) https://BioRender.com/gnn2qib, accessed on 20 April 2026.

Cardiovascular disease (CVD) continues to be a major worldwide health burden, significantly increasing mortality and disability (Figure 3) (Table 5). The World Health Organization estimates that CVD causes around 18 million deaths a year, or over one-third of all deaths worldwide. But the effects of CVD go beyond death; it results in long-term incapacity and rising healthcare costs, both of which severely lower patients’ and their families’ quality of life [73]. Despite the availability of numerous therapeutic alternatives, such as lipid-lowering drugs, antihypertensive medications, and cardiac surgical procedures, several significant obstacles still need to be overcome. These include a lack of novel treatments, poor patient adherence, delayed diagnosis, and unequal access to healthcare. When taken as a whole, these characteristics make it more difficult to effectively prevent, treat, and manage cardiovascular disease over the long term [74]. Emerging therapies, such as nucleic acid therapeutics and cell-based treatments, seek to solve these constraints by focusing on the underlying causes of disease, allowing for more accurate and successful interventions [75].

SiRNAs, which are intended to suppress the expression of target mRNAs, were the first RNA-based medications authorized for clinical use. Among these, the FDA and European Commission approved Patisiran (ONPATTRO^®^), a first-in-class RNA interference therapy that targets transthyretin to treat hereditary transthyretin-mediated amyloidosis with cardiomyopathy, an autosomal dominant disorder that severely impairs heart function [76]. Building on the success of RNA interference, mRNA-based treatments are currently being intensively investigated in several cardiovascular contexts, such as hereditary cardiomyopathies, heart failure, regenerative cardiology, and lipid diseases. These new approaches take advantage of mRNA’s special ability to control protein expression, providing promising paths for tissue healing and disease modification in cardiovascular medicine.

**Table 5:** The application of mRNA technology in cardiovascular diseases.

Condition	Route of Administration and Carrier	Therapeutic Effects	Model	References
Myocardial infarction.	Intramyocardial injection	Promote vascular and heart regeneration	Mouse	[77]
	Intravenous infusion, LNP	Targeting of the cardiac infarct zone	Mouse	[78]
	Intravenous infusion, RGD-PEG-PLGA NPs	Inhibition of cardiomyocyte apoptosis, and inflammation	Rat	[79]
	Intravenous infusion, Hep@PGEA NPs	Targeted damage to the heart	Mouse	[80]
	Intravenous infusion, LNP	Improving cardiac regeneration	Rat, Pig	[81]
	Epicardial injection, LNP	Demonstrated safety and tolerability.	Phase II clinical trial	[82]
Heart failure	Intravenous infusion, LNP	Reduction in cardiac fibrosis	Mouse	[83]
	Intravenous infusion, LNP	Reduction in cardiac fibrosis	Mouse	[84]
	Intravenous infusion	Hormone replacement therapy	Phase I clinical trial	[85]
	Intramyocardial injection	Promote angiogenesis and reduce fibrosis	Pig	[86]
	Inhalation, NPs	Inhibit cardiac myocyte hypertrophy and reduce fibrosis	Mouse	[87]
Atherosclerosis	Intravenous infusion, NP’s	Reduce the chronic inflammatory response	Mouse	[88]
	Intravenous infusion, LNP	The PCSK9 levels were significantly reduced	Mouse	[89]
	Subcutaneous injection	Reduce triglycerides	Advanced clinical	[90]
	Intravenous infusion, NP’s	Reduce the inflammatory response	Mouse	[91]
	Intravenous infusion, NP’s	Modulate inflammation in advanced atherosclerotic lesions	Mouse	[92]
Congenital Heart Disease	Intrauterine injection, LNP	Reduce cardiac toxicity	Mouse	[93]
Myocardiosis	Intravenous infusion, rBV	Anti-inflammatory action	Mouse	[94]
	Intravenous infusion, AAV-9	Prevent the development of cardiac hypertrophy	Mouse	[95]
	Intravenous infusion, AAV-9	Prevent the development of a dilated cardiomyopathy	Mouse	[96]
	Coronary sinus infusion, AAV-9	Diffuse myocardial transduction	Pig	[97]
	Intravenous infusion, LNP	The concentration of reducing TTR in the serum	Phase III clinical trial	[98]

### 4.3. Rare Genetic Diseases

Due to their low frequency and few available treatments, rare genetic diseases—also known as orphan diseases—have long presented serious problems for our healthcare system [99]. mRNA therapy has become a very promising treatment option for several diseases brought on by genetic abnormalities in recent years [100]. Using carriers such as lipid-based nanoparticles (LNPs), chemically altered mRNA is injected into cells to produce functional proteins that make up for genetic deficits [101]. mRNA therapies can safely and effectively fix genetic flaws in uncommon disorders and alleviate symptoms because of the benefits of precision dosing, biocompatibility, temporary expression, and low risk of genomic integration [102]. Numerous mRNA medications that target uncommon disorders are currently in clinical studies. Research advancements in a number of rare disease models and therapeutic trials are summarized in Table 6.

## 5. Safety and Immunogenicity

Key elements of LNPs, ionizable lipids, are essential for improving the transport and effectiveness of endosomal escape and cytosolic release of mRNAs. However, because of their role in immunology, energy metabolism, and cellular signaling, their possible toxicity needs to be carefully considered. Pro-inflammatory cytokines can be produced when they activate TLRs, especially TLR4 [103]. Ionizable lipids such as DLin-MC3-DMA and C12-200 have been shown to have an immunostimulatory effect. Ionizable lipid-derived metabolites, including fatty acids, cause toxicity by triggering peroxisome proliferator-activated receptors (PPARs). Inflammation and liver damage may arise from the activation of these mechanisms [104]. Through hepatic neutrophil infiltration, empty LNPs containing the ionizable lipid YSK13 have been demonstrated to increase plasma levels of aspartate aminotransferase (AST) and alanine aminotransferase (ALT), indicators of liver injury [105]. PEGylated lipids are another source of toxicity; their capacity to change the pharmacokinetics and biodistribution of LNPs raises questions about their long-term safety [104]. When PEGylated LNPs are administered repeatedly, the immune system may react and produce anti-PEG antibodies. Through faster blood clearance, these antibodies can quickly remove future doses of PEGylated LNPs from the circulation [106].

Lysosomal cysteine proteases, like Cathepsin B/D, can cause toxicity and inflammation in addition to lipids. After LNPs are endocytosed, these proteases are released into the cytosol during lysosomal membrane permeabilization (LMP), which triggers inflammation by activating the NLRP3 inflammasome and promoting the production of pro-inflammatory cytokines. Additionally, cathepsins cause cellular toxicity by triggering apoptosis pathways, rupturing the plasma membrane, and encouraging necroptosis and cell death [107,108]. LNP’s cargo, mRNA, can result in toxicities as well as off-target effects, innate immune activation, and protein overexpression. Off-target delivery causes antigens to be expressed by non-antigen-presenting cells, which can lead to unwanted immune reactions and tissue-specific harm such as myocarditis. Furthermore, naked mRNA is inherently unstable and can generate fragmented RNA species, thereby compromising translational integrity as it undergoes hydrolytic degradation [109]. These pieces have the ability to function as DAMPs, which can lead to further immunological activation and toxicity, excessive cytokine production, and systemic inflammation [104].

Research revealed that compared to typical LNPs, LNPs containing trehalose glycolipids demonstrated noticeably less toxicity in several organs, including the liver and heart. Another strategy is to substitute polysarcosine (pSar), which is derived from the endogenous amino acid sarcosine, for PEGylated lipids [36]. These lower the complement activation and pro-inflammatory cytokine secretion, reducing the risk of hypersensitivity reactions and complement activation-related pseudo-allergy. Higher protein expression and a better safety profile are made possible by this PEG-free method, which makes pSar-functionalized LNPs a potential platform with lower toxicity [36].

The way that LNP interacts with the immune system is influenced by its shape and charge, among other physical and chemical characteristics. The body’s immune system recognizes LNPs as foreign, triggering off innate immunity and then influencing adaptive immunity [110]. It triggers the generation of cytokines and systemic inflammation by activating innate immune sensors like TLR7, TLR8, and MDA5. Therefore, to optimize therapeutic advantages and reduce potential side effects, we want LNPs to excite these pathways to a manageable degree while balancing their activation. Previous research demonstrated that, irrespective of MAVS-mediated RNA-sensing pathways, LNP, as an integrated adjuvant, induces strong antigen-specific CD4^+^ TFH cell and GC B-cell responses by inducing IL-6 production [111].

While it is ideal for vaccines to elicit robust immune responses, reducing the immunogenicity of LNPs should also be considered, particularly when repeated administration is involved. Adverse immunological reactions, including severe allergic reactions and autoimmune manifestations, can be triggered by elevated immunogenicity, weakening the effectiveness of treatment and jeopardizing patient safety [112]. Strategies include modifying the composition and physicochemical characteristics of LNPs to overcome these issues. This entails adjusting the molar ratios of PEGylated lipids, phospholipids, cholesterol, and ionizable lipids. PEGylated lipids prolong circulation duration and improve LNP stability, but they can also cause the previously stated anti-PEG antibodies, which can trigger immunological responses. Therefore, this risk can be effectively reduced by using biodegradable polymers or cleavable PEG versions [113]. Additionally, it is crucial to optimize the size and surface charge of the nanoparticles; smaller, neutrally charged particles have better lymph node targeting and less immunogenicity. Immunogenic profiles are further refined by the addition of particular adjuvants that alter immune responses and the deliberate choice of administration routes, such as IV, IM, subcutaneous (SC), intradermal (ID), or intranasal (IN) [114].

## 6. Scalability and Manufacturing

The clinical translation for mRNA nanomedicines was shifted from vaccines to a broader range of applications after the COVID pandemic. However, they are very preliminary towards rare diseases, protein replacement, and oncology combinations. Based on data from ClinicalTrials.gov, 557 mRNA clinical trials were identified, of which around 91% are focused solely on vaccines, and the remaining 9% are split between non-vaccine applications [115]. Based on recent studies, microfluidics has now transitioned from a research tool to an industrial strategy, overcoming batch variability, fouling, and limited throughput. A study by Hwang et al. demonstrated that antifouling lubricant coating on the mixers of microfluidics reduces channel-based fouling as it maintains ideal particle size and high encapsulation. This enables the continuous operation even at higher flow rates while maintaining the product quality [116]. Their work directly addresses the barrier that prevents microfluidics at the industrial level. In addition to this study, Shepherd et al. have reported a ‘throughput-scalable microfluidic system’ which can produce SARS-CoV-2 LNP-mRNA vaccines that demonstrate the tunability of engineering and mixing kinetics to maintain the physicochemical properties as well as the formulation fidelity, facilitating its scalability [117]. Moreover, there is a need for high-quality mRNA synthesis based on the end-to-end protocols that are validated. Upstream-downstream integration is extremely essential to develop the next-generation platforms that pose different purities of the carrier chemistry or the structural requirements of the mRNA cargo. Leighton et al. have standardized the upstream parameters like IVT optimization, dsRNA removal, and capping efficiency to ensure their compatibility with the downstream [118]. Parallelly, the industry now sees a development of the process-compatible delivery materials, which are designed specifically for manufacturing rather than their biological performance. Recently, ionizable polymers and polymeric nanoparticles have been developed with self-assembly kinetics that align with the flow of lipid nanoparticle manufacturing structure. Wan et al. highlight that the controlled tertiary amine density and hydrophobic domain tuning enable the mRNA complexation and endosomal escape while enhancing their thermal stability and simplifying their precursor sourcing [119].

With COVID, there were several structural weaknesses found in the global mRNA manufacturing, including the reliance on specialized lipids and limited surge capacity. With the recent developments, decoupling mRNA from the delivery vehicle complexity is necessary for having workflows that are adaptable across the majority of the therapeutics. Rapid pivoting between the vaccine and therapeutics demonstrates the emerging need in industrial frameworks for LNP assembly as a modular operation [120]. Within recent years, the defining trend is the co-optimization of carrier chemistry and manufacturing architecture. The polymeric nanoparticles exhibit similar assembly behaviors, such as being sensitive to shear rate, solvent polarity, and mixing energy, which can easily be controlled in microfluidics, thus making them ideal as the next-generation candidates for continuous-flow systems [121]. This raises the need for the development of a manufacturing ecosystem where both material properties and process parameters can be co-designed for enabling diverse adaptation without the need for excessive revalidation. This unified platform-level scalability allows the polymer-based carriers to have the cross-compatible assembly pipelines, thus minimizing the downtime.

Together, these represent the transition towards a manufacturing ecosystem where material design, process control, and regulatory expectations are constructed as one framework. The future is working on the coherence of the entire production architecture. This ensures that mRNA therapeutics meet the demands with robustness.

## 7. Scientific Gaps-Untapped Potential of mRNA Therapeutics

Lipid nanoparticle (LNP) technology for RNA delivery has advanced from proof-of-concept to worldwide deployment in less than ten years. Onpattro’s 2018 clearance proved that LNPs could transport nucleic acids to tissues safely and efficiently. The COVID-19 pandemic demonstrated the mRNA–LNP vaccines’ scalability, versatility, and transformational potential by their quick development and widespread distribution. There are currently over 150 RNA-LNP formulations undergoing clinical studies. However, the great majority (more than 80%) focus on infectious diseases and cancer. Applications in acute critical illnesses (ACIs), which are among the leading causes of death worldwide, are uncommon. These include myocardial infarction, stroke, acute respiratory infections, and other severe injuries. Given that many ACIs have characteristics that might make them especially responsive to mRNA–LNP treatments, this disparity is unexpected [122].

Because they may be designed to carry various RNA cargos, mRNA–LNPs are especially well suited for these environments, allowing for the simultaneous regulation of multiple disease pathways. They are capable of producing intracellular proteins (or peptides), including those that would otherwise be unavailable to small molecules or conventional biologics. In individuals with multi-system organ dysfunction, organ-specific targeted techniques may lessen systemic exposure and toxicity. Furthermore, since most pharmacological advances are recombinant proteins that are more than 20 years old, like tissue plasminogen activator, there is little competition among platform technologies for ACIs. The nearest authorized rivals are peptides, which are straightforward but have issues with intracellular delivery and pharmacokinetics [122].

The lack of knowledge on the long-term immunogenicity of LNPs is one of the major obstacles. There is a significant knowledge gap regarding the long-term effects and safety of LNPs, especially for therapies requiring repeated administrations like those for chronic diseases and genetic disorders, as current research primarily focuses on the short-term immune responses and immediate impacts on vaccine efficacy. The necessity for thorough long-term studies that can clarify the possibility of immunological tolerance, chronic inflammation, and the overall effects of repeated doses over prolonged durations is highlighted by this gap.

The immunogenicity of LNPs continues to be a major safety concern. Although there is ample evidence of negative impacts, the underlying mechanisms are still poorly understood, and current research has not yet produced comprehensive solutions to reduce these risks. In-depth mechanistic research and the development of safer LNP formulations that can reduce these side effects without sacrificing therapeutic efficacy are therefore necessary. Additionally, there has not been much research done on different LNP administration methods. The effectiveness and safety of other possible methods, such as IN or SC injections, are not well studied, even though intramuscular and intravenous routes are frequently employed. Although they are still unexplored, these alternate paths may have clear benefits. To fully comprehend these paths’ possible advantages and disadvantages, future studies should investigate them in greater detail.

### Approaches to Increase Stability

Preventing physical and chemical degradation are two important areas to concentrate on in order to improve the stability of mRNA delivery devices. In order to safeguard these systems from mechanical and thermal stresses, stabilizing chemicals or protective coatings must be applied. In order to effectively prevent premature degradation, these preventative measures are carefully designed to maintain the system’s integrity under a variety of physical situations [123]. Notably, it has been demonstrated that the buffering species selected for the formulation is crucial and may enhance the stability of RNA pharmaceuticals, particularly when it comes to LNP/RNA drug products. It is also crucial to address chemical deterioration. This entails adding customized chemical changes to the delivery system that are intended to resist enzymatic activity and environmental elements that could otherwise jeopardize stability. For example, changing the cholesterol content of particular systems can stabilize lipid layers, encouraging the cohesiveness and liquid-ordered phases of lipids [124].

Nowadays, the most popular method for dealing with and preventing the aforementioned types of deterioration in long-term storage is freeze-drying, which has been shown to extend the shelf life of pharmaceutical items by eliminating water [125]. Research has demonstrated that adding cooling agents and cryoprotectants like trehalose and sucrose can preserve the effectiveness of LNPs during freeze–thaw cycles. This is important because it tackles the problem of preserving stability in LNPs under conditions like freezing and thawing, which are typical for transportation [126]. However, relying solely on freezing these goods to preserve stability is unsatisfactory because of their expensive cost, as well as transportation and accessibility issues when the treatments must be kept in refrigerated conditions.

Techniques to prevent the oxidation of the mRNA ribose nucleobases and the hydrolysis of the mRNA phosphodiester backbone are essential. The stability of the mRNA within the delivery system might be improved by using chemically altered nucleotides to lessen hydrolysis susceptibility [127].

Chemical modification of the mRNA, such as the addition of pseudouridine, which can also improve the stability of the mRNA, may boost the resistance to hydrolysis even further. In essence, the pseudouridine prevents the innate immune system from identifying the mRNA molecule as alien, thereby averting immune system-induced destruction. Redesigning RNA molecules to create double-stranded sections is another efficient method of reducing mRNA hydrolysis [128]. This structural change preserves the capacity to code for the desired proteins while offering defense against in-line cleavage and enzymatic degradation. Additionally, adding antioxidants to the mixture, such as glutathione and ascorbic acid, creates a barrier that guards against oxidative stress and maintains the structural integrity of mRNA.

At the same time, hybrid nanoparticles, which combine the advantages of both organic and inorganic materials onto a single platform have come to the forefront. These nanoparticles usually have a core-shell structure, in which the organic shell increases biocompatibility and improves targeted delivery capabilities while the inorganic core provides structural resilience and regulated release dynamics [129].

Additionally, core-shell shaped lipopolyplex nanoparticles and nanostructured lipid carriers, which are essential parts of several mRNA COVID-19 vaccines, have been approved for use in humans in several different parts of the world. This includes the Gemcovac^®^-19 vaccine from Gennova Biopharma (Pune, India), which was authorized for use in 2022, and the SW-BIC-213^®^ vaccine from Stemirna Therapeutics (Shanghai, China), which is presently undergoing a phase 3 clinical trial [130]. In lyophilized powder form or at refrigerated temperatures, these delivery systems have been reported to be stable and bioactive for over several months [131]. In addition to emphasizing stability, the interaction of organic and inorganic components in these hybrid structures allows for the modification of release profiles, a crucial feature for increasing the therapeutic effect of mRNA therapies [132].

Together, these strategies aid in the creation of reliable mRNA delivery systems that can continue to function well in therapeutic settings.

## 8. Future Directions and Conclusions

The development of mRNA treatments will be fueled by the synergistic integration of several cutting-edge fields, including improved clinical precision, deeper mechanistic understanding, scalable data analytics, and extended delivery options, rather than by separate discoveries. The shift from small-scale discovery to systematic, high-throughput investigation of mRNA structural elements (5′ cap, UTRs, poly-A tail) within extensive molecular landscapes is crucial to this advancement. The batch-effect inconsistencies that currently impede cross-platform reproducibility (e.g., discrepancies between Moderna, Acuitas, and Protiva datasets) could be overcome by foundation models pre-trained on extensive datasets of ionizable lipids, polymers, and hybrid nanoparticles, followed by institution-specific fine-tuning. Diversifying delivery beyond hepatic dominance is a crucial challenge. Targeting ligands found using diffusion-based generative models trained on high-resolution imaging data will be necessary to achieve organ-selective tropism (e.g., lung, spleen, CNS). AI-driven formulation design must simultaneously optimize LNPs for different routes of administration (intranasal, intradermal, and intrathecal), each of which presents distinct biophysical limitations (ionic strength, mucosal barriers, immune-microenvironment interactions). Lastly, AI might predict epitope immunogenicity and co-optimize LNP chemistry to match the patient’s immune-metabolic profile when designing neoantigen vaccines for oncology based on a patient’s tumor phylogeny. 262 chemically altered mRNAs could be contained in LNPs designed to avoid tissue-specific microRNA-mediated suppression in protein replacement therapies, reducing off-target immune activation [133]. When taken as a whole, these developments hint at a future in which automated microfluidic devices would swiftly synthesize mRNA therapeutics once they have been computationally designed, refined, and customized. This will replace conventional trial-and-error processes with a precision-driven, high-throughput paradigm.

Collectively, these directions provide a trajectory for mRNA therapeutics, which is not only a vaccine technology but a major therapeutic backbone whose clinical reach is broadened by nanotechnology. With the integration of carrier designs and attention to safety, these approaches have the potential to move from case studies to the treatment of rare diseases.

## Figures and Tables

**Table 2:** Characterization matrix for the design parameters.

Design Parameter	Measurement Method	Target Metric	Performance Impact	References
Ionizable lipid pKa	Thioflavin T fluorescence titration	6.2–6.9	Endosomal escape	[30]
PEG lipid shedding	Serum Incubation	1–4 h half life	Cellular uptake efficiency	[25]
Nitrogen to phosphate ratio	RiboGreen assay with detergent lysis	More than 90% encapsulation	Ribonuclease protection	[37]
Nanoparticle size and Poly dispersity Index (PDI)	Dynamic Light Scattering and nanoparticle tracking	50–150 nm with PDI under 0.2	Pharmacokinetics and biodistribution	[38]
Internal Morphology	Cryo-transmission electron microscopy	Lipid phase formation at pH 5.5	Membrane fusion capacity	[39]

**Table 3:** Comparative analysis of emerging mRNA platforms with relative advantages, persistent limitations, and critical research gaps.

Platform	Advantages	Key Limitations	Research Needs	References
Biodegradable Ionizable Lipids	Faster Clearance, Lower liver accumulation	Ester hydrolysis reduces stability and degradation unclear	GMP catalyst-free synthesis	[61,62,63]
Charge-Switching Lipids	Microenvironment responsive and enhanced mucus penetration	Triggers heterogeneity across tumors, standardized enzyme assay unavailable	Correlation for clinical trigger efficacy	[17,61,64]
Polymeric Carriers	Tunable targeting	10–100× lower encapsulation	Trials with direct LNPs in disease models	[65,66]
Biomimetic Systems	Tissue-specific uptake	Scale up Challenges, Batch heterogeneity	Standardized purification, GMP protocols	[67,68]

**Table 4:** List of clinical trials for mRNA-based cancer vaccines by data compiled from ClinicalTrials.gov (accessed on 23 January 2026) using unfiltered advanced search with keywords “mRNA” AND (“oncology” OR “cancer” OR ‘’tumor”).

Cancer	Sponsor	Phase and NCT Number	Intervention	Discussion of Results Obtained from the Trial
Brain tumor	Guangdong 999 Brain Hospital	Phase 1 NCT02808416	mRNA tumor antigen pulsed DC	Personalized DC vaccines encoding patient-specific TAA panels were given to ten patients and were well-tolerated, with no Grade III/IV toxicities observed. Among seven evaluable patients, most developed antigen-specific CD4^+^ and/or CD8^+^ T-cell responses, and overall survival exceeded that of standard-of-care patients at the same center, without signs of autoimmune toxicity.
	Oslo University Hospital	Phase 1, Phase 2 NCT00846456	Intradermal injection of transfected dendritic cells	Autologous CSC cultures and high-quality mRNA were successfully generated from most tumors, enabling DC vaccination in seven patients. All vaccinated patients mounted immune responses without autoimmune or other significant toxicity, and progression-free survival was markedly longer than in matched controls (694 vs. 236 days). These data indicate that CSC-targeted vaccination for glioblastoma is safe, well-tolerated, and may prolong progression-free survival.
	University of Florida	Phase 1 NCT03396575	Adoptive cellular therapy combined with dendritic cell vaccines, administered during recovery from focal radiotherapy.	No update in literature as of 2026
	CureVac (Tübingen, Germany)	Phase 1 NCT05938387	CV09050101 mRNA vaccine	Personalized mRNA vaccine targeting multiple glioblastoma TAAs, given IM after surgery/RT. Dose-escalation data show safety, no DLTs, and de novo CD4+/CD8^+^ T-cell responses in ~77% of patients at 100 µg dose (selected for expansion). No full paper; conference abstracts only as of 2026.
Gastrointestinal tumor	Merck Sharp & Dohme LLC (Rahway, NJ, USA)	Phase 1 NCT03948763	V941 (mRNA-5671/V941) as a monotherapy and in combination with pembrolizumab infusion	A strategic program discontinuation with no published results available.
	National Cancer Institute (NCI)	Phase 1, Phase 2 NCT03480152	NCI-4650	Personalized KRAS^G12D^ neoepitope mRNA vaccine was safe in 4 patients, eliciting de novo mutation-specific T-cell responses and isolatable TCRs, though no objective responses were seen. Future combinations with checkpoint inhibitors or ACT recommended.
	Gritstone bio, Inc. (Emeryville, Ca, USA)	Phase 2 NCT05456165	Individualized neoantigen vaccine called GRT-C901/GRT-R902 (chimpanzee adenovirus [ChAd] and self-amplifying messenger RNA	Personalized KRAS^G12D^ neoepitope mRNA vaccine was safe in 4 patients, eliciting de novo mutation-specific T-cell responses and isolatable TCRs, though no objective responses were seen. Future combinations with checkpoint inhibitors or ACT recommended.
Lung cancer	Ludwig Institute for Cancer Research	Phase 1, Phase 2 NCT03164772	mRNA Vaccine BI 1361849	No update in literature as of 2026
Solid tumors	ModernaTX, Inc. (Cambridge, MA, USA)	Phase 1 NCT03739931	mRNA-2752	As per conference abstracts in 2026, mRNA-2752 injection with durvalumab was pharmacodynamically active, inducing ≥2-fold systemic IL-23 and IFN-γ increases, higher CD8 T-cell abundance/proliferation, and upregulation of immune-inflamed gene signatures in tumor tissue, particularly in patients with complete/partial response or stable disease.
Hematologic malignancy	M.D. Anderson Cancer Center	Phase 1 NCT00514189	dendritic cells loaded with acute myelogenous leukemia lysate plus mRNA	No update in literature as of 2026
	Memorial Sloan Kettering Cancer Center	Phase 1 NCT01995708	WT1 mRNA-electroporated Autologous Langerhans-type Dendritic Cells	Posttransplant LC-DC vaccine (CT7/MAGE-A3/WT1 mRNA) with lenalidomide maintenance was safe (no > grade 1 toxicity), induced antigen-specific CD4/CD8 T-cell cytokine secretion (IFN-γ, IL-2, TNF-α) and CD107a upregulation, with clonal T-cell expansion trends favoring vaccinated patients.
Melanoma	Oslo University Hospital	Phase 1, Phase 2NCT01278940	mRNA-Transfected Dendritic Cells	Autologous DC vaccine transfected with tumor mRNA was feasible and safe in 22 advanced melanoma patients (no serious adverse events). Immune responses seen in 9/19 (T-cell assays) and 8/18 (delayed-type hypersensitivity); intradermal route superior to intranodal (7/10 vs. 3/12 responders).
	Radboud University Medical Center	Phase 1, Phase 2 NCT00243529	Peptide-pulsing or mRNA Transfection	Phase II peptide-pulsed DC vaccine (HLA-A24/A2-restricted gp100, tyrosinase, MAGEs, MART-1) safe in 24 metastatic melanoma patients (no > grade III adverse events). Immune responses (ELISPOT 75%, delayed-type hypersensitivity) and pre-vaccine anti-melanoma antibody titers correlated with prolonged overall survival.
	Steinar Aamdal	Phase 1, Phase 2 NCT00961844	Tumor Cell-Derived mRNA	Terminated early due to logistical issues; no published results available
	Radboud University Medical Center	Phase 1, Phase 2 NCT01530698	TLR-DC and Trimix DC	No published results available as of 2026
	BioNTech SE (Mainz, Germany)	Phase 2 NCT04526899	BNT111	No published results available as of 2026
	Genentech, Inc. (South San Francisco, CA, USA)	Phase 2 NCT03815058	Autogene cevumeran	No published results available as of 2026
Ovarian Cancer	Steinar Aamdal	Phase 1 NCT01334047	Autologous Dendritic Cells Loaded With Amplified Ovarian Cancer Stem Cell mRNA, hTERT and Survivin.	The study needed to be terminated due to new knowledge about cancer vaccines. A new protocol with an expected more efficient vaccine is currently being written.
	University Medical Center Groningen	Phase 1 NCT04163094	Liposome Formulated mRNA Vaccine in Combination With Neo-Adjuvant Chemotherapy	No published results available as of 2026

**Table 6:** Clinical trials of mRNA therapies for rare genetic diseases.

Condition	Sponsor	Phase and NCT Number	Intervention	Discussion of Results Obtained from the Trial
Propionic Acidemia	ModernaTX, Inc.	Phase 1, Phase 2 NCT04159103	mRNA-3927 delivered via LNPs	Sixteen participants across five dose cohorts received 346 intravenous doses over 15.7 person-years of exposure. No dose-limiting toxicities occurred, with treatment-emergent adverse events in 94% of participants. Among eight participants with metabolic decompensation events in the prior 12 months, risk decreased by 70% during treatment.
Propionic Acidemia	ModernaTX, Inc.	Phase 1, Phase 2 NCT05130437	mRNA-3927 delivered via LNPs	No published results available as of 2026
Familial Hypercholesterolemia	Tang-Du Hospital	Phase 1, NCT05043181	LDLR mRNA delivered via exosomes	No published results available as of 2026
Cystic Fibrosis	Vertex Pharmaceuticals Incorporated (Boston, MA, USA)	Phase 1, NCT05668741	VX-522 delivered via LNPs	No published results available as of 2026
Cystic Fibrosis	Translate Bio, Inc. (Lexington, MA, USA)	Phase 1, Phase 2 NCT03375047	MRT-5005 delivered via LNPs	Forty-two participants received MRT5005 (31) or placebo (11). Fourteen mild-to-moderate febrile reactions occurred in 10 MRT5005-treated patients; two discontinued due to fever. Two hypersensitivity reactions occurred and resolved with conservative management. Cough and headache were most common adverse events. Forced expiratory volume in one second remained stable but showed no treatment benefit
Cystic Fibrosis	Arcturus Therapeutics, Inc. (San Diego, CA, USA)	Phase 1, NCT0571253	ARCT-032 delivered via LUNAR LNPs	Single ascending doses safe and well-tolerated in 32 healthy volunteers and 4 cystic fibrosis patients; minimal systemic lipid exposure (less than 1 nanogram per milliliter). Improved mucociliary clearance observed in cystic fibrosis patients; no percent predicted forced expiratory volume in 1 s efficacy data reported.
Glycogen Storage Disease	ModernaTX, Inc.	Phase 1, NCT05095727	mRNA-3745 delivered via LNPs	No published results available as of 2026
Glycogen Storage Disease Type III	Ultragenyx Pharmaceutical Inc. (Novato, CA, USA)	Phase 1, Phase 2 NCT04990388	UX053 delivered via LNPs	Terminated but not because of safety concerns.
Ornithine Transcarbamylase Deficiency	Arcturus Therapeutics, Inc.	Phase 1, NCT04442347	ARCT-810 delivered via LUNAR LNPs	No published results available as of 2026
Ornithine Transcarbamylase Deficiency	Arcturus Therapeutics, Inc.	Phase 2, NCT05526066	ARCT-810 delivered via LUNAR LNPs	No published results available as of 2026
Ornithine Transcarbamylase Deficiency	Arcturus Therapeutics, Inc.	Phase 1, NCT04416126	ARCT-810 delivered via LUNAR LNPs	ARCT-810 well-tolerated at therapeutic doses; all adverse events mild or moderate with no serious events reported. Favorable pharmacokinetics shown; no lipid detectable in plasma beyond 48 h post-administration.
Ornithine Transcarbamylase Deficiency	Translate Bio, Inc.	Phase 1, Phase 2 NCT03767270	MRT5201 delivered via LNPs	Program discontinued
Methylmalonic Acidemia	ModernaTX, Inc.	Phase 1, Phase 2 NCT05295433	mRNA-3705 delivered via LNPs	No published results available as of 2026
Methylmalonic Acidemia	ModernaTX, Inc.	Phase 1, Phase 2 NCT04899310	mRNA-3705 delivered via LNPs	No published results available as of 2026
Methylmalonic Acidemia	ModernaTX, Inc.	Phase 1, Phase 2 NCT03810690	mRNA-3704 delivered via LNPs	Study was terminated due to a business decision and not due to safety or efficacy reasons

## Data Availability

No new data were created or analyzed in this study. Data sharing is not applicable to this article.

## References

[1] Forenzo C., Larsen J. (2023). Complex Coacervates as a Promising Vehicle for mRNA Delivery: A Comprehensive Review of Recent Advances and Challenges. Mol. Pharm..

[2] Viegas C., Seck F., Fonte P. (2022). An insight on lipid nanoparticles for therapeutic proteins delivery. J. Drug Deliv. Sci. Technol..

[3] Hald Albertsen C., Kulkarni J.A., Witzigmann D., Lind M., Petersson K., Simonsen J.B. (2022). The role of lipid components in lipid nanoparticles for vaccines and gene therapy. Adv. Drug Deliv. Rev..

[4] Huang X., Kong N., Zhang X., Cao Y., Langer R., Tao W. (2022). The landscape of mRNA nanomedicine. Nat. Med..

[5] Yadav N., Debnath N., Das S. (2026). Stimuli-responsive nanocarriers for targeted mRNA therapeutics: A paradigm shift in mRNA delivery for biomedical applications. Drug Deliv. Transl. Res..

[6] Puccetti M., Pariano M., Schoubben A., Ricci M., Giovagnoli S. (2024). Engineering carrier nanoparticles with biomimetic moieties for improved intracellular targeted delivery of mRNA therapeutics and vaccines. J. Pharm. Pharmacol..

[7] Ahl P.L. (2025). Microfluidic and Turbulent Mixing for mRNA LNP Vaccines. Pharmaceutics.

[8] Wu Y., Yu S., De Lázaro I. (2024). Advances in lipid nanoparticle mRNA therapeutics beyond COVID-19 vaccines. Nanoscale.

[9] Elliott L., Foster T., Castillo P., Mendez-Gomez H., Sayour E.J. (2025). Therapeutic mRNA vaccine applications in oncology. Mol. Ther..

[10] Lv Z., Dai Y. (2025). mRNA vaccines and SiRNAs targeting cancer immunotherapy: Challenges and opportunities. Discov. Oncol..

[11] Gawalski K., Przybyszewska W., Hunia J., Gawalska A., Rymarz A. (2025). Unraveling the potential: mRNA therapeutics in oncology. Front. Oncol..

[12] Younis M.A., Sato Y., Kimura S., Harashima H. (2025). A new strategy for the extrahepatic delivery of lipid-based nanomedicines: A protein corona-mediated selective targeting system based on an ionizable cationic lipid library. RSC Pharm..

[13] Fang Z., Monteiro V.S., Oh C., Janabi K.A., Romero L., Ahsan N., Yang L., Peng L., DiMaio D., Lucas C. (2025). A modular vaccine platform for optimized lipid nanoparticle mRNA immunogenicity. Nat. Biomed. Eng..

[14] Abughalia A., Flynn M., Clarke P.F.A., Fayne D., Gobbo O.L. (2025). The Use of Computational Approaches to Design Nanodelivery Systems. Nanomaterials.

[15] Zhao S., Chen J., Dai T., Li G., Huang L., Xin J., Zhang Y., Chen Y., He X., Huang H. (2025). Harnessing Computational Strategies to Overcome Challenges in mRNA Vaccines. Physiology.

[16] Hou X., Zaks T., Langer R., Dong Y. (2021). Lipid nanoparticles for mRNA delivery. Nat. Rev. Mater..

[17] Li X., Li J., Wei J., Du W., Su C., Shen X., Zhao A., Xu M. (2025). Design Strategies for Novel Lipid Nanoparticle for mRNA Vaccine and Therapeutics: Current Understandings and Future Perspectives. MedComm.

[18] Zhao Y., Wang Z.M., Song D., Chen M., Xu Q. (2024). Rational design of lipid nanoparticles: Overcoming physiological barriers for selective intracellular mRNA delivery. Curr. Opin. Chem. Biol..

[19] Han X., Zhang H., Butowska K., Swingle K.L., Alameh M.-G., Weissman D., Mitchell M.J. (2021). An ionizable lipid toolbox for RNA delivery. Nat. Commun..

[20] Wei P.-S., Thota N., John G., Chang E., Lee S., Wang Y., Ma Z., Tsai Y.-H., Mei K.-C. (2024). Enhancing RNA-lipid nanoparticle delivery: Organ- and cell-specificity and barcoding strategies. J. Control. Release.

[21] Sabnis S., Kumarasinghe E.S., Salerno T., Mihai C., Ketova T., Senn J.J., Lynn A., Bulychev A., McFadyen I., Chan J. (2018). A Novel Amino Lipid Series for mRNA Delivery: Improved Endosomal Escape and Sustained Pharmacology and Safety in Non-human Primates. Mol. Ther..

[22] Maier M.A., Jayaraman M., Matsuda S., Liu J., Barros S., Querbes W., Tam Y.K., Ansell S.M., Kumar V., Qin J. (2013). Biodegradable Lipids Enabling Rapidly Eliminated Lipid Nanoparticles for Systemic Delivery of RNAi Therapeutics. Mol. Ther..

[23] Gao P. (2025). PEGylated lipids in lipid nanoparticle delivery dynamics and therapeutic innovation. Beilstein J. Nanotechnol..

[24] Paunovska K., Loughrey D., Dahlman J.E. (2022). Drug delivery systems for RNA therapeutics. Nat. Rev. Genet..

[25] Mui B.L., Tam Y.K., Jayaraman M., Ansell S.M., Du X., Tam Y.Y.C., Lin P.J., Chen S., Narayanannair J.K., Rajeev K.G. (2013). Influence of Polyethylene Glycol Lipid Desorption Rates on Pharmacokinetics and Pharmacodynamics of siRNA Lipid Nanoparticles. Mol. Ther.—Nucleic Acids.

[26] Sahay G., Alakhova D.Y., Kabanov A.V. (2010). Endocytosis of nanomedicines. J. Control. Release.

[27] Gilleron J., Querbes W., Zeigerer A., Borodovsky A., Marsico G., Schubert U., Manygoats K., Seifert S., Andree C., Stöter M. (2013). Image-based analysis of lipid nanoparticle–mediated siRNA delivery, intracellular trafficking and endosomal escape. Nat. Biotechnol..

[28] Cheng J., Jian L., Chen Z., Li Z., Yu Y., Wu Y. (2024). In Vivo Delivery Processes and Development Strategies of Lipid Nanoparticles. ChemBioChem.

[29] Kauffman K.J., Dorkin J.R., Yang J.H., Heartlein M.W., DeRosa F., Mir F.F., Fenton O.S., Anderson D.G. (2015). Optimization of Lipid Nanoparticle Formulations for mRNA Delivery in Vivo with Fractional Factorial and Definitive Screening Designs. Nano Lett..

[30] Hassett K.J., Benenato K.E., Jacquinet E., Lee A., Woods A., Yuzhakov O., Himansu S., Deterling J., Geilich B.M., Ketova T. (2019). Optimization of Lipid Nanoparticles for Intramuscular Administration of mRNA Vaccines. Mol. Ther.—Nucleic Acids.

[31] Cheng Q., Wei T., Farbiak L., Johnson L.T., Dilliard S.A., Siegwart D.J. (2020). Selective organ targeting (SORT) nanoparticles for tissue-specific mRNA delivery and CRISPR–Cas gene editing. Nat. Nanotechnol..

[32] Sarode A., Patel P., Vargas-Montoya N., Allawzi A., Zhilin-Roth A., Karmakar S., Boeglin L., Deng H., Karve S., DeRosa F. (2024). Inhalable dry powder product (DPP) of mRNA lipid nanoparticles (LNPs) for pulmonary delivery. Drug Deliv. Transl. Res..

[33] Xu X., Xia T. (2023). Recent Advances in Site-Specific Lipid Nanoparticles for mRNA Delivery. ACS Nanosci. Au.

[34] Chheda U., Pradeepan S., Esposito E., Strezsak S., Fernandez-Delgado O., Kranz J. (2024). Factors Affecting Stability of RNA—Temperature, Length, Concentration, pH, and Buffering Species. J. Pharm. Sci..

[35] Navid Talemi M., Ramezani Farani M., Alipour Eskandani N., Mirzaee D., Alipourfard I., Huh Y.S. (2026). Programmable lipid nanoparticles for RNA therapeutics: Design principles and clinical translation. Mater. Today Bio.

[36] Wang J., Ding Y., Chong K., Cui M., Cao Z., Tang C., Tian Z., Hu Y., Zhao Y., Jiang S. (2024). Recent Advances in Lipid Nanoparticles and Their Safety Concerns for mRNA Delivery. Vaccines.

[37] Schober G.B., Story S., Arya D.P. (2024). A careful look at lipid nanoparticle characterization: Analysis of benchmark formulations for encapsulation of RNA cargo size gradient. Sci. Rep..

[38] Dahlman J.E., Barnes C., Khan O.F., Thiriot A., Jhunjunwala S., Shaw T.E., Xing Y., Sager H.B., Sahay G., Speciner L. (2014). In vivo endothelial siRNA delivery using polymeric nanoparticles with low molecular weight. Nat. Nanotechnol..

[39] Rajesh S., Zhai J., Drummond C.J., Tran N. (2021). Synthetic ionizable aminolipids induce a pH dependent inverse hexagonal to bicontinuous cubic lyotropic liquid crystalline phase transition in monoolein nanoparticles. J. Colloid Interface Sci..

[40] Hosseini-Kharat M., Bremmell K.E., Prestidge C.A. (2025). Why do lipid nanoparticles target the liver? Understanding of biodistribution and liver-specific tropism. Mol. Ther. Methods Clin. Dev..

[41] Kim H.L., Saravanakumar G., Lee S., Jang S., Kang S., Park M., Sobha S., Park S.-H., Kim S.-M., Lee J.-A. (2025). Poly(β-amino ester) polymer library with monomer variation for mRNA delivery. Biomaterials.

[42] Wang H., Wang Y., Yuan C., Xu X., Zhou W., Huang Y., Lu H., Zheng Y., Luo G., Shang J. (2023). Polyethylene glycol (PEG)-associated immune responses triggered by clinically relevant lipid nanoparticles in rats. npj Vaccines.

[43] Dong S., Healy L., Gong F., Xu Y., Cai Y., Solek N.C., Chen J., Zhou M., Thomson T., Savguira M. (2026). Biodegradable lipid nanoparticles for genome editing in the brain via intrathecal administration. Mater. Today.

[44] Huang P., Deng H., Zhou Y., Chen X. (2022). The roles of polymers in mRNA delivery. Matter.

[45] Xu Y., Gong F., Golubovic A., Strilchuk A., Chen J., Zhou M., Dong S., Seto B., Li B. (2025). Rational design and modular synthesis of biodegradable ionizable lipids via the Passerini reaction for mRNA delivery. Proc. Natl. Acad. Sci. USA.

[46] Han X., Xu Y., Ricciardi A., Xu J., Palanki R., Chowdhary V., Xue L., Gong N., Alameh M.-G., Peranteau W.H. (2025). Plug-and-play assembly of biodegradable ionizable lipids for potent mRNA delivery and gene editing in vivo. bioRxiv.

[47] Chen Z., Tian Y., Yang J., Wu F., Liu S., Cao W., Xu W., Hu T., Siegwart D.J., Xiong H. (2023). Modular Design of Biodegradable Ionizable Lipids for Improved mRNA Delivery and Precise Cancer Metastasis Delineation In Vivo. J. Am. Chem. Soc..

[48] Polidori I., Weber L.I., Keim S., To D., Hartl M., Bernkop-Schnürch A. (2026). Alkaline phosphatase-triggered charge converting lipid nanoparticles: An innovative approach for oral nucleic acid delivery. Int. J. Pharm..

[49] Zöller K., Haddadzadegan S., Lindner S., Veider F., Bernkop-Schnürch A. (2024). Design of charge converting lipid nanoparticles via a microfluidic coating technique. Drug Deliv. Transl. Res..

[50] Yang F., Lei L., Wang X., Hong J., Wu Z., Jiang J.-H. (2024). Engineering Cell-Selective Charge-Altering Lipid Nanoparticles for Efficient siRNA Delivery in Vivo. CCS Chem..

[51] Trines M.M., Hoorn D., Hofstraat S.R.J., Zwolsman R.C., Anbergen T., Versteeg I., Van Elsas Y., Deckers J., Hendrikx M.M.A., Kleuskens T. (2026). Dendrimers Improve Apolipoprotein Nanoparticle mRNA Delivery to Immune Cells. Adv. Mater..

[52] Meshanni J.A., Stevenson E.R., Zhang D., Sun R., Ona N.A., Reagan E.K., Abramova E., Guo C.-J., Wilkinson M., Baboo I. (2025). Targeted delivery of TGF-β mRNA to murine lung parenchyma using one-component ionizable amphiphilic Janus Dendrimers. Nat. Commun..

[53] Yong H., Tian Y., Li Z., Wang C., Zhou D., Liu J., Huang X., Li J. (2025). Highly Branched Poly(β-amino ester)s for Efficient mRNA Delivery and Nebulization Treatment of Silicosis. Adv. Mater..

[54] Pilipenko I., Korovkina O., Gubina N., Ekimova V., Ishutinova A., Korzhikova-Vlakh E., Tennikova T., Korzhikov-Vlakh V. (2022). Random Copolymers of Lysine and Isoleucine for Efficient mRNA Delivery. Int. J. Mol. Sci..

[55] Garcia B.B.M., Douka S., Mertins O., Mastrobattista E., Han S.W. (2024). Efficacy of Chitosan-N-Arginine Chitosomes in mRNA Delivery and Cell Viability Enhancement. ACS Appl. Bio Mater..

[56] Liu H., Deng Y., Li J., Lin W., Liu C., Yang X., Zhou Z., Jiang Y. (2025). Ginger-derived exosome-like nanoparticles: A representative of plant-based natural nanostructured drug delivery system. Front. Bioeng. Biotechnol..

[57] Tang Z., You X., Xiao Y., Chen W., Li Y., Huang X., Liu H., Xiao F., Liu C., Koo S. (2023). Inhaled mRNA nanoparticles dual-targeting cancer cells and macrophages in the lung for effective transfection. Proc. Natl. Acad. Sci. USA.

[58] Sahay G., Querbes W., Alabi C., Eltoukhy A., Sarkar S., Zurenko C., Karagiannis E., Love K., Chen D., Zoncu R. (2013). Efficiency of siRNA delivery by lipid nanoparticles is limited by endocytic recycling. Nat. Biotechnol..

[59] Kulkarni J.A., Witzigmann D., Thomson S.B., Chen S., Leavitt B.R., Cullis P.R., Van Der Meel R. (2021). The current landscape of nucleic acid therapeutics. Nat. Nanotechnol..

[60] Iqbal Z., Rehman K., Mahmood A., Shabbir M., Liang Y., Duan L., Zeng H. (2024). Exosome for mRNA delivery: Strategies and therapeutic applications. J. Nanobiotechnol..

[61] Zhai J., Cote T., Chen Y. (2024). Challenges and advances of the stability of mRNA delivery therapeutics. Nucleic Acid Insights.

[62] Nabhan J.F., Wood K.M., Rao V.P., Morin J., Bhamidipaty S., LaBranche T.P., Gooch R.L., Bozal F., Bulawa C.E., Guild B.C. (2016). Intrathecal delivery of frataxin mRNA encapsulated in lipid nanoparticles to dorsal root ganglia as a potential therapeutic for Friedreich’s ataxia. Sci. Rep..

[63] Akinc A., Maier M.A., Manoharan M., Fitzgerald K., Jayaraman M., Barros S., Ansell S., Du X., Hope M.J., Madden T.D. (2019). The Onpattro story and the clinical translation of nanomedicines containing nucleic acid-based drugs. Nat. Nanotechnol..

[64] Eftekhari Z., Zohrabi H., Oghalaie A., Ebrahimi T., Shariati F.S., Behdani M., Kazemi-Lomedasht F. (2024). Advancements and challenges in mRNA and ribonucleoprotein-based therapies: From delivery systems to clinical applications. Mol. Ther.—Nucleic Acids.

[65] Yang W., Mixich L., Boonstra E., Cabral H. (2023). Polymer-Based mRNA Delivery Strategies for Advanced Therapies. Adv. Healthc. Mater..

[66] Zhao F., Luppi B., Chao P.-H., Yang J., Zhang Y., Feng R., Chan V., Kannan R., Dong S., Gogoulis A. (2026). Biodegradable polymers with tertiary amines enhance mRNA delivery of lipid nanoparticles via improved endosomal escape. Biomaterials.

[67] Yin M., Sun H., Li Y., Zhang J., Wang J., Liang Y., Zhang K. (2024). Delivery of mRNA Using Biomimetic Vectors: Progress and Challenges. Small.

[68] Kim H.I., Park J., Zhu Y., Wang X., Han Y., Zhang D. (2024). Recent advances in extracellular vesicles for therapeutic cargo delivery. Exp. Mol. Med..

[69] Li Y., Wang M., Peng X., Yang Y., Chen Q., Liu J., She Q., Tan J., Lou C., Liao Z. (2023). mRNA vaccine in cancer therapy: Current advance and future outlook. Clin. Transl. Med..

[70] Fournier C., Mercey-Ressejac M., Derangère V., Al Kadi A., Rageot D., Charrat C., Leroy A., Vollaire J., Josserand V., Escudé M. (2025). Nanostructured lipid carriers based mRNA vaccine leads to a T cell–inflamed tumour microenvironment favourable for improving PD-1/PD-L1 blocking therapy and long-term immunity in a cold tumour model. eBioMedicine.

[71] Li R., Hu J.-C., Rong L., He Y., Wang X., Lin X., Li W., Wu Y., Kuwentrai C., Su C. (2025). The guided fire from within: Intratumoral administration of mRNA-based vaccines to mobilize memory immunity and direct immune responses against pathogen to target solid tumors. Cell Discov..

[72] Ben-Akiva E., Karlsson J., Hemmati S., Yu H., Tzeng S.Y., Pardoll D.M., Green J.J. (2023). Biodegradable lipophilic polymeric mRNA nanoparticles for ligand-free targeting of splenic dendritic cells for cancer vaccination. Proc. Natl. Acad. Sci. USA.

[73] Vaduganathan M., Mensah G.A., Turco J.V., Fuster V., Roth G.A. (2022). The Global Burden of Cardiovascular Diseases and Risk. J. Am. Coll. Cardiol..

[74] Steinmetz J.D., Seeher K.M., Schiess N., Nichols E., Cao B., Servili C., Cavallera V., Cousin E., Hagins H., Moberg M.E. (2024). Global, regional, and national burden of disorders affecting the nervous system, 1990–2021: A systematic analysis for the Global Burden of Disease Study 2021. Lancet Neurol..

[75] Argiro A., Bui Q., Hong K.N., Ammirati E., Olivotto I., Adler E. (2024). Applications of Gene Therapy in Cardiomyopathies. JACC Heart Fail..

[76] Hoy S.M. (2018). Patisiran: First Global Approval. Drugs.

[77] Zangi L., Lui K.O., Von Gise A., Ma Q., Ebina W., Ptaszek L.M., Später D., Xu H., Tabebordbar M., Gorbatov R. (2013). Modified mRNA directs the fate of heart progenitor cells and induces vascular regeneration after myocardial infarction. Nat. Biotechnol..

[78] Evers M.J.W., Du W., Yang Q., Kooijmans S.A.A., Vink A., Van Steenbergen M., Vader P., De Jager S.C.A., Fuchs S.A., Mastrobattista E. (2022). Delivery of modified mRNA to damaged myocardium by systemic administration of lipid nanoparticles. J. Control. Release.

[79] Sun Y.-S., Zhao Z., Yang Z.-N., Xu F., Lu H.-J., Zhu Z.-Y., Shi W., Jiang J., Yao P.-P., Zhu H.-P. (2017). Risk Factors and Preventions of Breast Cancer. Int. J. Biol. Sci..

[80] Nie J., Qiao B., Duan S., Xu C., Chen B., Hao W., Yu B., Li Y., Du J., Xu F. (2018). Unlockable Nanocomplexes with Self-Accelerating Nucleic Acid Release for Effective Staged Gene Therapy of Cardiovascular Diseases. Adv. Mater..

[81] Turnbull I.C., Eltoukhy A.A., Fish K.M., Nonnenmacher M., Ishikawa K., Chen J., Hajjar R.J., Anderson D.G., Costa K.D. (2016). Myocardial Delivery of Lipidoid Nanoparticle Carrying modRNA Induces Rapid and Transient Expression. Mol. Ther..

[82] Anttila V., Saraste A., Knuuti J., Hedman M., Jaakkola P., Laugwitz K.-L., Krane M., Jeppsson A., Sillanmäki S., Rosenmeier J. (2023). Direct intramyocardial injection of VEGF mRNA in patients undergoing coronary artery bypass grafting. Mol. Ther..

[83] Tang J., Wang J., Huang K., Ye Y., Su T., Qiao L., Hensley M.T., Caranasos T.G., Zhang J., Gu Z. (2018). Cardiac cell–integrated microneedle patch for treating myocardial infarction. Sci. Adv..

[84] Rurik J.G., Tombácz I., Yadegari A., Méndez Fernández P.O., Shewale S.V., Li L., Kimura T., Soliman O.Y., Papp T.E., Tam Y.K. (2022). CAR T cells produced in vivo to treat cardiac injury. Science.

[85] Shi Y., Shi M., Wang Y., You J. (2024). Progress and prospects of mRNA-based drugs in pre-clinical and clinical applications. Signal Transduct. Target. Ther..

[86] Collén A., Bergenhem N., Carlsson L., Chien K.R., Hoge S., Gan L.-M., Fritsche-Danielson R. (2022). VEGFA mRNA for regenerative treatment of heart failure. Nat. Rev. Drug Discov..

[87] Weng H., Zou W., Tian F., Xie H., Liu A., Liu W., Liu Y., Zhou N., Cai X., Wu J. (2024). Inhalable cardiac targeting peptide modified nanomedicine prevents pressure overload heart failure in male mice. Nat. Commun..

[88] Krienke C., Kolb L., Diken E., Streuber M., Kirchhoff S., Bukur T., Akilli-Öztürk Ö., Kranz L.M., Berger H., Petschenka J. (2021). A noninflammatory mRNA vaccine for treatment of experimental autoimmune encephalomyelitis. Science.

[89] Liu J., Chang J., Jiang Y., Meng X., Sun T., Mao L., Xu Q., Wang M. (2019). Fast and Efficient CRISPR/Cas9 Genome Editing In Vivo Enabled by Bioreducible Lipid and Messenger RNA Nanoparticles. Adv. Mater..

[90] Chebli J., Larouche M., Gaudet D. (2024). APOC3 siRNA and ASO therapy for dyslipidemia. Curr. Opin. Endocrinol. Diabetes Obes..

[91] Gao C., Huang Q., Liu C., Kwong C.H.T., Yue L., Wan J.-B., Lee S.M.Y., Wang R. (2020). Treatment of atherosclerosis by macrophage-biomimetic nanoparticles via targeted pharmacotherapy and sequestration of proinflammatory cytokines. Nat. Commun..

[92] Gao M., Tang M., Ho W., Teng Y., Chen Q., Bu L., Xu X., Zhang X.-Q. (2023). Modulating Plaque Inflammation via Targeted mRNA Nanoparticles for the Treatment of Atherosclerosis. ACS Nano.

[93] Gao K., Li J., Song H., Han H., Wang Y., Yin B., Farmer D.L., Murthy N., Wang A. (2023). In utero delivery of mRNA to the heart, diaphragm and muscle with lipid nanoparticles. Bioact. Mater..

[94] Lasrado N., Reddy J. (2020). An overview of the immune mechanisms of viral myocarditis. Rev. Med. Virol..

[95] Mearini G., Stimpel D., Geertz B., Weinberger F., Krämer E., Schlossarek S., Mourot-Filiatre J., Stoehr A., Dutsch A., Wijnker P.J.M. (2014). Mybpc3 gene therapy for neonatal cardiomyopathy enables long-term disease prevention in mice. Nat. Commun..

[96] Xie C., Zhang Y.-P., Song L., Luo J., Qi W., Hu J., Lu D., Yang Z., Zhang J., Xiao J. (2016). Genome editing with CRISPR/Cas9 in postnatal mice corrects PRKAG2 cardiac syndrome. Cell Res..

[97] Myers V.D., Landesberg G.P., Bologna M.L., Semigran M.J., Feldman A.M. (2022). Cardiac Transduction in Mini-Pigs After Low-Dose Retrograde Coronary Sinus Infusion of AAV9-BAG3. JACC Basic Transl. Sci..

[98] Kotit S. (2023). Lessons from the first-in-human in vivo CRISPR/Cas9 editing of the TTR gene by NTLA-2001 trial in patients with transthyretin amyloidosis with cardiomyopathy. Glob. Cardiol. Sci. Pract..

[99] Shen G., Liu J., Yang H., Xie N., Yang Y. (2024). mRNA therapies: Pioneering a new era in rare genetic disease treatment. J. Control. Release.

[100] Rohner E., Yang R., Foo K.S., Goedel A., Chien K.R. (2022). Unlocking the promise of mRNA therapeutics. Nat. Biotechnol..

[101] Qin S., Tang X., Chen Y., Chen K., Fan N., Xiao W., Zheng Q., Li G., Teng Y., Wu M. (2022). mRNA-based therapeutics: Powerful and versatile tools to combat diseases. Signal Transduct. Target. Ther..

[102] Baptista B., Carapito R., Laroui N., Pichon C., Sousa F. (2021). mRNA, a Revolution in Biomedicine. Pharmaceutics.

[103] Parhiz H., Brenner J.S., Patel P.N., Papp T.E., Shahnawaz H., Li Q., Shi R., Zamora M.E., Yadegari A., Marcos-Contreras O.A. (2022). Added to pre-existing inflammation, mRNA-lipid nanoparticles induce inflammation exacerbation (IE). J. Control. Release.

[104] Bitounis D., Jacquinet E., Rogers M.A., Amiji M.M. (2024). Strategies to reduce the risks of mRNA drug and vaccine toxicity. Nat. Rev. Drug Discov..

[105] Sato Y., Matsui H., Yamamoto N., Sato R., Munakata T., Kohara M., Harashima H. (2017). Highly specific delivery of siRNA to hepatocytes circumvents endothelial cell-mediated lipid nanoparticle-associated toxicity leading to the safe and efficacious decrease in the hepatitis B virus. J. Control. Release.

[106] Ishida T., Kiwada H. (2008). Accelerated blood clearance (ABC) phenomenon upon repeated injection of PEGylated liposomes. Int. J. Pharm..

[107] Yadati T., Houben T., Bitorina A., Shiri-Sverdlov R. (2020). The Ins and Outs of Cathepsins: Physiological Function and Role in Disease Management. Cells.

[108] Xie Z., Zhao M., Yan C., Kong W., Lan F., Narengaowa, Zhao S., Yang Q., Bai Z., Qing H. (2023). Cathepsin B in programmed cell death machinery: Mechanisms of execution and regulatory pathways. Cell Death Dis..

[109] Cheng F., Wang Y., Bai Y., Liang Z., Mao Q., Liu D., Wu X., Xu M. (2023). Research Advances on the Stability of mRNA Vaccines. Viruses.

[110] Lee J., Woodruff M.C., Kim E.H., Nam J.-H. (2023). Knife’s edge: Balancing immunogenicity and reactogenicity in mRNA vaccines. Exp. Mol. Med..

[111] Alameh M.-G., Tombácz I., Bettini E., Lederer K., Sittplangkoon C., Wilmore J.R., Gaudette B.T., Soliman O.Y., Pine M., Hicks P. (2022). Lipid nanoparticles enhance the efficacy of mRNA and protein subunit vaccines by inducing robust T follicular helper cell and humoral responses. Immunity.

[112] Li B., Jiang A.Y., Raji I., Atyeo C., Raimondo T.M., Gordon A.G.R., Rhym L.H., Samad T., MacIsaac C., Witten J. (2023). Enhancing the immunogenicity of lipid-nanoparticle mRNA vaccines by adjuvanting the ionizable lipid and the mRNA. Nat. Biomed. Eng..

[113] Xu H., Wang K.Q., Deng Y.H., Chen D.W. (2010). Effects of cleavable PEG-cholesterol derivatives on the accelerated blood clearance of PEGylated liposomes. Biomaterials.

[114] Anderluzzi G., Lou G., Woods S., Schmidt S.T., Gallorini S., Brazzoli M., Johnson R., Roberts C.W., O’Hagan D.T., Baudner B.C. (2022). The role of nanoparticle format and route of administration on self-amplifying mRNA vaccine potency. J. Control. Release.

[115] Li Q., Zeng M., Lv W., Ye J., Wu S. (2026). Current landscape of clinical trials for mRNA-based therapeutics. Hum. Vaccines Immunother..

[116] Hwang Y.-H., Shepherd S.J., Kim D., Mukalel A.J., Mitchell M.J., Issadore D.A., Lee D. (2025). Robust, Scalable Microfluidic Manufacturing of RNA–Lipid Nanoparticles Using Immobilized Antifouling Lubricant Coating. ACS Nano.

[117] Shepherd S.J., Han X., Mukalel A.J., El-Mayta R., Thatte A.S., Wu J., Padilla M.S., Alameh M.-G., Srikumar N., Lee D. (2023). Throughput-scalable manufacturing of SARS-CoV-2 mRNA lipid nanoparticle vaccines. Proc. Natl. Acad. Sci. USA.

[118] Leighton L.J., Chaudhary N., Tompkins H.T., Kulkarni A., Carrodus N.L., Budzinska M.A., Lakshman Das S., Cheetham S.W., Mercer T.R. (2025). The design, manufacture and LNP formulation of mRNA for research use. Nat. Protoc..

[119] Wan Q., Sun Y., Sun X., Zhou Z. (2024). Rational design of polymer-based mRNA delivery systems for cancer treatment. Polym. Chem..

[120] Youssef M., Hitti C., Puppin Chaves Fulber J., Kamen A.A. (2023). Enabling mRNA Therapeutics: Current Landscape and Challenges in Manufacturing. Biomolecules.

[121] Yousefi Adlsadabad S., Hanrahan J.W., Kakkar A. (2024). mRNA Delivery: Challenges and Advances through Polymeric Soft Nanoparticles. Int. J. Mol. Sci..

[122] (2025). The untapped potential of mRNA–lipid nanoparticles. Nat. Rev. Bioeng..

[123] Packer M., Gyawali D., Yerabolu R., Schariter J., White P. (2021). A novel mechanism for the loss of mRNA activity in lipid nanoparticle delivery systems. Nat. Commun..

[124] Briuglia M.-L., Rotella C., McFarlane A., Lamprou D.A. (2015). Influence of cholesterol on liposome stability and on in vitro drug release. Drug Deliv. Transl. Res..

[125] Kasper J.C., Winter G., Friess W. (2013). Recent advances and further challenges in lyophilization. Eur. J. Pharm. Biopharm..

[126] Stark B., Pabst G., Prassl R. (2010). Long-term stability of sterically stabilized liposomes by freezing and freeze-drying: Effects of cryoprotectants on structure. Eur. J. Pharm. Sci..

[127] Karikó K., Buckstein M., Ni H., Weissman D. (2005). Suppression of RNA Recognition by Toll-like Receptors: The Impact of Nucleoside Modification and the Evolutionary Origin of RNA. Immunity.

[128] Wayment-Steele H.K., Kim D.S., Choe C.A., Nicol J.J., Wellington-Oguri R., Watkins A.M., Parra Sperberg R.A., Huang P.-S., Participants E., Das R. (2021). Theoretical basis for stabilizing messenger RNA through secondary structure design. Nucleic Acids Res..

[129] Andretto V., Repellin M., Pujol M., Almouazen E., Sidi-Boumedine J., Granjon T., Zhang H., Remaut K., Jordheim L.P., Briançon S. (2023). Hybrid core-shell particles for mRNA systemic delivery. J. Control. Release.

[130] Yang R., Deng Y., Huang B., Huang L., Lin A., Li Y., Wang W., Liu J., Lu S., Zhan Z. (2021). A core-shell structured COVID-19 mRNA vaccine with favorable biodistribution pattern and promising immunity. Signal Transduct. Target. Ther..

[131] Gerhardt A., Voigt E., Archer M., Reed S., Larson E., Van Hoeven N., Kramer R., Fox C., Casper C. (2022). A flexible, thermostable nanostructured lipid carrier platform for RNA vaccine delivery. Mol. Ther.—Methods Clin. Dev..

[132] Siewert C.D., Haas H., Cornet V., Nogueira S.S., Nawroth T., Uebbing L., Ziller A., Al-Gousous J., Radulescu A., Schroer M.A. (2020). Hybrid Biopolymer and Lipid Nanoparticles with Improved Transfection Efficacy for mRNA. Cells.

[133] Ige M.A., Ren X., Yang Y., Zhang H., Shen C., Jiang Y., Li J., Wan X. (2025). mRNA therapeutics: Transforming medicine through innovation in design, delivery, and disease treatment. Mol. Ther. Nucleic Acids.

